# Spawning Site Selection and Contingent Behavior in Common Snook, *Centropomus undecimalis*

**DOI:** 10.1371/journal.pone.0101809

**Published:** 2014-07-07

**Authors:** Susan Lowerre-Barbieri, David Villegas-Ríos, Sarah Walters, Joel Bickford, Wade Cooper, Robert Muller, Alexis Trotter

**Affiliations:** 1 Florida Fish and Wildlife Conservation Commission, Florida Fish and Wildlife Research Institute, St. Petersburg, Florida, United States of America; 2 Department of Ecology and Marine Resources, Institute of Marine Research (IIM-CSIC), Vigo, Pontevedra, Spain; North Carolina State University, United States of America

## Abstract

Reproductive behavior affects spatial population structure and our ability to manage for sustainability in marine and diadromous fishes. In this study, we used fishery independent capture-based sampling to evaluate where Common Snook occurred in Tampa Bay and if it changed with spawning season, and passive acoustic telemetry to assess fine scale behavior at an inlet spawning site (2007–2009). Snook concentrated in three areas during the spawning season only one of which fell within the expected spawning habitat. Although in lower numbers, they remained in these areas throughout the winter months. Acoustically-tagged snook (n = 31) showed two seasonal patterns at the spawning site: Most fish occurred during the spawning season but several fish displayed more extended residency, supporting the capture-based findings that Common Snook exhibit facultative catadromy. Spawning site selection for iteroparous, multiple-batch spawning fishes occurs at the lifetime, annual, or intra-annual temporal scales. In this study we show colonization of a new spawning site, indicating that lifetime spawning site fidelity of Common Snook is not fixed at this fine spatial scale. However, individuals did exhibit annual and intra-seasonal spawning site fidelity to this new site over the three years studied. The number of fish at the spawning site increased in June and July (peak spawning months) and on new and full lunar phases indicating within population variability in spawning and movement patterns. Intra-seasonal patterns of detection also differed significantly with sex. Common Snook exhibited divergent migration tactics and habitat use at the annual and estuarine scales, with contingents using different overwintering habitat. Migration tactics also varied at the spawning site at the intra-seasonal scale and with sex. These results have important implications for understanding how reproductive behavior affects spatio-temporal patterns of fish abundance and their resilience to disturbance events and fishing pressure.

## Introduction

Improving knowledge of stock structure and life cycle processes in diadromous and marine fishes is resulting in new understanding of factors affecting productivity and how to manage for sustainability [1]–[6]. Two commonly used conceptual models to address complex stock structure are the metapopulation concept [7], [8] and contingent theory [9]–[11], which both focus on spatial distribution of behavioral groups and their ultimate relationship to reproductive isolation. However, metapopulation analysis more commonly addresses persistence or extinction of spawning groups, whereas a contingent is defined as “a discrete segment of a population that diverges spatially along an alternative migratory pathway during the course of life history” [11]. The majority of recent research on contingents has been based on early life history and otolith microchemistry, but with the advent of acoustic telemetry, it is now possible to track individuals over time [12]. Assessing spatio-temporal patterns in reproductive behavior is becoming more common but results from these studies need to be integrated into concepts of population structure. This is especially important for highly fecund marine species as they typically support important fisheries, exhibit poor stock-recruitment relationships, and their spatio-temporal reproductive behavior may impact productivity as much as, or more than, adult stock size [13].

Key spatial elements of an individual's life cycle include: where an individual is spawned (i.e., the spawning site used by its parents) larval retention area, juvenile nursery habitat, adult feeding habitat and spawning site selection, which closes the life cycle and results in either philopatry or allopatry [14]. Understanding the range and overlap between adult feeding and spawning grounds and individual variability in how these areas are used over time have important implications for assessing spawning site selection, spawning migrations, and the potential for sex to define contingent spatial behavior. Equally important is an understanding of fine scale spatio-temporal reproductive behavior, as this determines the first environment all fish encounter, affecting later life cycle processes and ultimately population structure.

The Common Snook, *Centropomus undecimalis*, is a catadromous, subtropical species, exhibiting a protandric hermaphroditic gender system and an extended spawning season from mid-April through mid-September in the Gulf of Mexico [15]. It is highly targeted as a game fish in Florida waters, supporting an economically important fishery [16], [17]. The long-held paradigm has been that adult snook use river habitat as thermal refuge during winter months [18] and in the spring and summer move to higher salinity spawning habitat (> 24 ‰) to ensure buoyancy of fertilized eggs [19]–[21]. However, recent research suggests that spatial dynamics of adult snook are more complex than previously believed, with some adults remaining in the rivers during spawning months [22] while others overwinter in the estuaries [22], [23]. On the east coast of Florida, where there are fewer inlets and passes, snook form large aggregations in these locations year after year, sustaining high levels of catch-and-release fishing [16], [24], [25]. On the west coast of Florida, Common Snook exhibit strong spawning site fidelity [26], [27], although spawning aggregations appear to be smaller and more dispersed, with spawning occurring at numerous passes and inlets at the mouths of estuaries and along adjacent beaches [26]–[29].

In this study, we used capture-based survey data at the estuarine scale, as well as acoustic telemetry of individuals at a recently colonized spawning site, to evaluate both large and fine scale spatial dynamics of Common Snook in Tampa Bay, Florida. These data were used to test the following hypotheses: (1) spatial trends in Common Snook abundance will differ between spawning and non-spawning seasons; (2) Common Snook will be concentrated during the spawning season in high-salinity habitat near the mouth of Tampa Bay; (3) Common Snook will exhibit falcultative catadromy with a contingent remaining in the estuary year-round; (4) Common Snook will exhibit spawning site fidelity at both intra- and inter-annual temporal scales; and (5) the probability of an individual occurring at this spawning site within the expected window of spawning activity (seasonal and diel) will be affected by sex, sex-specific size, date within the spawning season, and lunar phase.

## Material and Methods

### Ethics

No specific permission for sampling was required, as sampling was conducted by the Florida Fish and Wildlife Conservation Commission's Fish and Wildlife Research Institute. However, every effort was made to meet all ethical standards (see below the methods used to decrease stress in acoustically tagged fishes). No protected species were sampled.

### Study location and period

**Figure 1:**
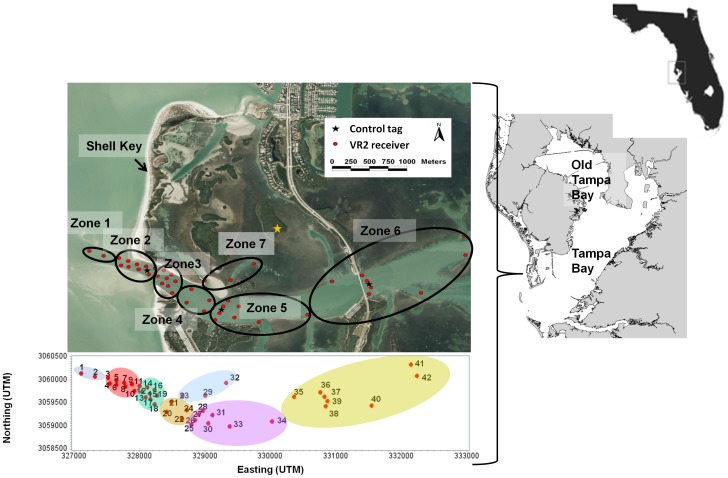
Maps of the receiver array and Tampa Bay. Location of Tampa Bay in southwest Florida and the receiver array located at an inlet spawning site at the mouth of Tampa Bay. Receiver locations are denoted with a red dot and control tag locations with a black star. Detection zones are indicated by black ovals. The number of each receiver, its relative position, and zone is indicated below the map. Receivers 3–19 (Zones 2 and 3) monitored the spawning site and are considered the core array. A gold star indicates Sawyer Key, where a fish was recaptured in January 2008.

This study was conducted at two spatial scales: in Tampa Bay, Florida's largest open-water estuary, and at an inlet spawning site at the mouth of Tampa Bay (Fig 1). Tampa Bay is shallow (3.7 m average depth) and extends over 1,000 km^2^ with numerous freshwater tributaries. Mangroves dominate much of Tampa Bay's coastline, especially on the eastern shore. The inlet study site was chosen because, in 2006, large numbers of actively spawning Common Snook were observed along the beach on the southern shore of Shell Key (Lowerre-Barbieri, unpubl. data), which forms the northern edge of the inlet (Fig. 1). The inlet is approximately 300 m across, with a relatively deep channel (maximum depth 8.5 m) bordered by sand bars. The bottom is predominantly shell hash, with no submerged aquatic vegetation or oyster reefs and currents are tidally-driven and can reach a maximum of 1 m/s (1.9 knots), decreasing as one moves eastward toward the estuary. Individual snook (n = 31) sampled along the northern edge of the inlet were acoustically tagged in June 2007 (*see details below*) and passively monitored with a receiver array deployed at the inlet and nearby areas. Twenty fish had tags that lasted 149 d and eleven fish had three-year tags that remained active throughout the 2008 and 2009 spawning seasons.

### Capture-based sampling

Estuarine-wide Fishery-Independent Monitoring (FIM) of Tampa Bay has been conducted monthly since 1996 using a 183-m haul seine and a stratified, random sampling design. Sampling sites were selected from a sampling universe consisting of one nautical mile square grids throughout Tampa Bay, which included the inlet spawning site (see below). Tampa Bay was divided into sampling zones based on logistical and hydrological characteristics and each zone stratified into areas by habitat. Monthly sampling was conducted at sites randomly selected from the strata available for each zone [30]. Data recorded included: abundance (including zeros, i.e. those hauls that did not catch Common Snook), location and size, as well as salinity, water temperature and the presence of bottom vegetation. We assessed adult fish from this data set by selecting individuals with a standard length (SL) ≥350 mm to be consistent with Winner et al. [29], but this is considered conservative as Taylor et al. reported 50% maturity at 250 mm total length (201 mm SL) [28].

Although the inlet spawning site was part of the FIM universe, additional sampling was conducted here to assess reproductive behavior. To evaluate the temporal pattern of snook presence and spawning at this site, fish were captured with a 122-m knotless center bag haul seine with 0.6 cm mesh, approximately weekly from April through June. The haul seine was set along the beach, corresponding to the northern edge of the core array (*see Receiver Array below*). Prior to setting the net, we drove the length of the beach to make visual observations of fish and/or courtship behavior. Weather permitting, two net sets were made per date and the net was retrieved immediately after deployment. Salinity was measured each sampling date. Spawning activity of fish captured at the inlet from April to June 2007 (n = 56) was determined based on successful strip-spawning of males and ovarian biopsies of females. Ovarian tissue was processed as follows: fixed in 10% neutrally buffered formalin for 24 h, soaked in water for 24 h, and stored in 70% ethanol. Samples were embedded in glycol methacrylate, sectioned to 3–5-mm thickness, stained with periodic acid–Schiff's hematoxylin, and then counterstained with metanil yellow [31]. Reproductive phases were assigned following the criteria in Brown-Peterson et al. [32].

### Receiver array

We deployed an array of 42 acoustic receivers (VR2s, Vemco Ltd, Shad Bay, NS, Canada) to monitor the inlet and nearby areas (Fig. 1). The array was an extension of that described in Lowerre-Barbieri et al. 2013 [33] and designed so that a lack of detections in the core array (Zones 2 and 3) corresponded to dates when fish were not at the spawning site. The core array consisted of seventeen receivers with overlapping ranges deployed off the beach where Common Snook spawned. Long-term range testing indicated that 85 m was the maximum range corresponding to consistent detection (> 50%) at this site due to tidal currents [33]. Receivers were arranged so that all paths through the core array were monitored by a minimum of three receivers. Another two receivers were moored in the Gulf of Mexico ≈ 200 m and ≈ 400 m west of the core array. The remaining 23 receivers were deployed nearby in the estuary (Fig. 1). Although this was not a VPS (Vemco positioning system), the use of control tags [33]–[35], allowed us to estimate position error [36] to determine if the resolution of our data was sufficient for the hypotheses tested. We deployed three control tags (69 kHz Vemco V9sc-2L 139 dB with a 60 s fixed delay) in different zones (see below) within the array (Fig. 1).

### Fish tagging

Because we wanted to use telemetry to assess spawning site fidelity, we did not implant any fish until spawning individuals were captured in the haul seine samples referenced above. Fish were sampled in the early evening (1735–2144 h) as this is the expected time of spawning [15]. To decrease stress the bag of the haul seine (2.4 m^3^) was kept submerged while by-catch items were removed. Individual snook were kept continuously immersed in transit from the net to the surgery station on the boat, by allowing them to swim into a plastic sling. On the boat, fish were kept in a live well (795 l capacity) until it was time for surgery. No more than five fish were held at a time. To ensure the fish's well-being, the live well was set up as a flow-through system, allowing fish to remain in ambient, oxygenated water [33]. Individuals selected for surgery were removed from the live well after they voluntarily swam into the sling.

A total of 31 snook (15F:16M) were intra-peritoneally implanted with Vemco coded transmitters. Eleven fish (5F:6M) were implanted with V13 tags (147 dB output, 540 d battery life, 30–90 s random inter-pulse delay) and 20 fish (10F:10M) were implanted with V9 tags (V9-2L-R, 146 dB output, 149 d battery life, 15–45 s random inter-pulse delay). Based on the expected spatio-temporal behavior of snook, the V13 tags were programmed to be active for 6 months (22 March through 20 September) to track fish during the spawning season (April-September [15]) and inactive the remaining six months to conserve battery life, allowing us to monitor snook over three spawning seasons.

Fish implanted in June 2007 ranged in size from 495 to 792 mm TL, with the average size of females (650 mm TL) being significantly larger than that of males (591 mm TL; two-tailed t-test, *n* = 31, *P* =  0.0308). Average surgery time was 7 m 20 s±2 m 40 s SD. The surgical procedure followed that of Lowerre-Barbieri et al.[33], with the exception that anesthesia was not used. Given that Aqui-S is no longer approved for use in the United States with food fish and these fish needed to be released immediately, an alternative method to calm fish during surgery was needed. Preliminary efforts indicated Common Snook responded well to surgery when fish were calmed by being turned ventral side up and having their eyes covered with a wet paper towel. Ambient, oxygenated water was flushed over their gills throughout the surgery. All males expressed milt on pressure and their reproductive phase was active spawning. Ovarian biopsies were taken from all females prior to surgery for histological analysis of reproductive state and the presence of spawning indicators [37], [38]. Biopsies were taken with a catheter composed of a 10 cc syringe equipped with an adapter and Tygon tubing with an inner diameter of 1.6 mm. The tubing was inserted 10–20 mm into the urogenital pore and the plunger of the syringe extended to create a vacuum to extract oocytes. This method has been shown to be effective for assessing reproductive state without the need for sacrificing the fish (Lowerre-Barbieri unpubl. data). After surgery, all fish received an external dart tag and were held in the live well for a minimum of 15 m before being released at the site of capture.

### Data analysis

**Figure 2:**
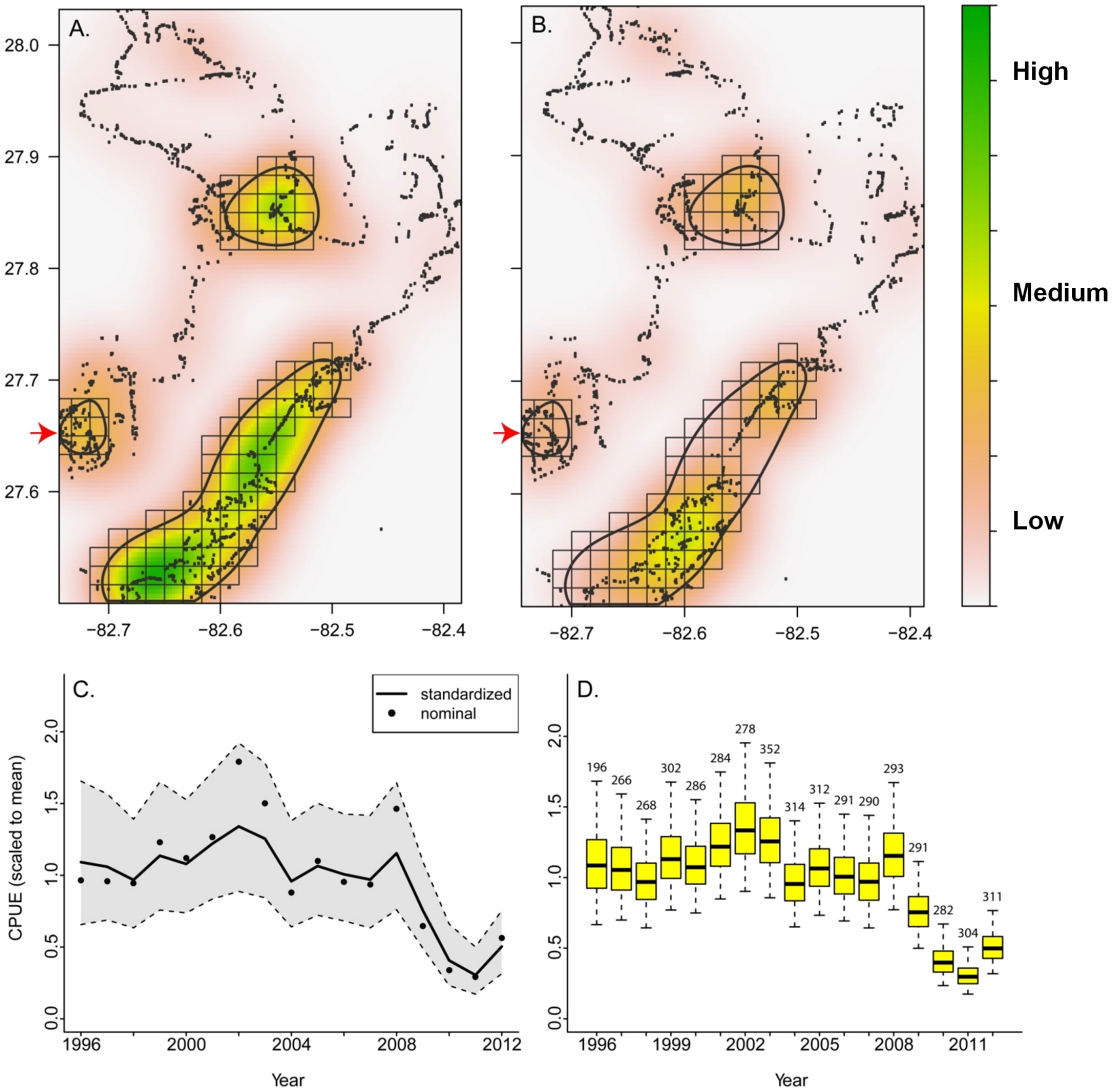
Spatial distribution and annual trends in catch of adult Common Snook. Snook were collected throughout Tampa Bay from 1996–2012 with fishery independent haul seine sets. Regions where snook were concentrated are denoted by color and the kernel density contour. All sampling grids within the contour were grouped to represent a concentrated region. The red arrow indicates the inlet spawning site. Each sampling site is denoted by a black dot. Regions with high concentrations of snook during the spawning season (A) and (B) outside the spawning season. (C) Trends in annual nominal and standardized CPUE and (D) distributions of standardized annual CPUE.

To understand the spatio-temporal patterns of snook estuary-wide, catch rates from the FIM data were assessed using a two-part hurdle model to account for zero-inflated data [39] with a generalized linear model (GLM). Before building the model, nominal CPUE data of snook ≥350 mm SL were plotted spatially to determine if there were areas where snook catches were concentrated in Tampa Bay and whether these areas differed with the spawning season. Based on this analysis, Tampa Bay was broken into two regions, one corresponding to high concentrations of snook and the other to low concentrations during the spawning season. These regions were spatially defined by performing a kernel density estimate (KDE) on the spatial distribution of nominal CPUE, selecting a contour of the KDE by which to classify high versus low concentrations, and assigning each sampling grid as either high versus low concentration (Fig. 2). The two-part GLM consisted of (1) a presence-absence model that estimated the proportion of hauls that caught snook (binomial distribution with a logit link) and (2) a presence-only model that estimated the number of snook ≥350 mm SL caught in an average successful haul (log-normal distribution with an identity link). Potential explanatory variables for both sub-models included year, month, region (concentrated or not), spawning season (within: April-September vs. outside: October-March), a region-by-spawning season interaction (i.e., to detect potential movements), temperature grouped into 2.5°C bins, salinity grouped into 2.5‰ bins, and presence/absence of bottom vegetation. The grouping of temperature and salinity was done to remove the linear constraint from the models. The final set of sub-models was chosen as the set with the lowest AIC using a stepwise forward-selection procedure. Year was retained in all final sub-models to obtain an index of abundance over time. Finally, year-specific, marginal means estimates and their standard errors from the two sub-models were used to generate distributions of estimates for each sub-model from a Monte Carlo simulation (10,000 Student's t distributed realizations) and the product of these estimates after back transforming from the log scales provided the distribution of the catch rate with year-specific variability. All analyses were done using R version 3.0.1 (R Development Core Team, 2011).

A database was developed in Access to integrate implantation and detection data of acoustically tagged fish, with times reported in Eastern Standard Time. Data were filtered to remove potential spurious detections (n = 29) which were defined as a single detection from a transmitter code (fish ID) in the core array over a 24 h period [40]. Positions were estimated using the weighted means method [41], [42] with an hourly time bin (mean detections per time bin equaled 9.6±0.09 SE) and based on two measures of location: either UTM coordinates or VR2 receiver numbers. To detect any abnormalities in behavior due to the stress of implantation [33], [36], data from the first 48 h (post-release period) were analyzed separately. The study period was considered to start after this initial 48 h. Two fish apparently died in 2009, i.e., they exhibited no movement over an extended time period: tag 26 stopped moving on 10 June 2009 and tag 30 stopped moving on 25 June 2009. Detections from these tags were removed from the data set after these dates, which resulted in 176,702 detections used for subsequent analysis.

To simplify spatial interpretation, fish positions based on the weighted means method were assigned to one of seven zones, similar to the approach of Danylchuk et al. [43]. Zones 1–6 were along the main channel to the inlet and within the estuary (Zone 1 being in the Gulf of Mexico and Zone 6 being the furthest within the estuary, Fig. 1). Zone 7 was an area of shallower water to the north of the main channel. The northern edge of Zones 2 and 3, along the beach, corresponds to where spawning activity was observed and actively spawning fish were sampled. Thus, the core array was developed to cover Zones 2 and 3, and these zones are referred to as the spawning site.

To assess the general spatial patterns of detected fish over the three years, we plotted hourly positions of all fish by year. To test whether the numbers of detections varied with zone, we used a Chi-squared test. Residence time was calculated based on annual total period of detection (TP, the number of days from the first detection to the last detection for a given tag in a year) [44], [45] and estimated separately for the spawning site and non-spawning zones. The number of days detected (DD) was also calculated. Residence indices (RI) were estimated for the array (A) and for the spawning site (SS) as the ratio of DD/TP for fish detected on five or more dates. We used a Pearson's correlation test to compare RI_A_ and RI_SS_ and a nonparametric Mann-Whitney test to assess differences by sex.

To model the probability of detection within the spawning site during the temporal window associated with spawning, we used a generalized additive mixed model (GAMM) with a binomial distribution (link = logit). Presence was coded as either 1 (present) or 0 (absent) to identify fish detected within Zones 2 and 3 during the spawning season and within the hours expected to correlate with moving to the site and spawning (1400–2000 h). Analyses were performed separately for all tagged fish in 2007 and for those fish with three-year tags across all years. Explanatory variables tested were: sex, sex-specific size (i.e., size was evaluated separately for males and females due to the protandric life history of Common Snook), detection date, and lunar phase, with year tested in the multi-year analysis. Sex was included as a categorical predictor and both day of the year and lunar phase were modeled with cyclic cubic regression splines. For lunar phase, the predictor was a continuous variable from 0 to 360 (full moon = 180, new moon = 0 and 360). The use of alternative spline bases (thin plate regression splines, cyclic P-splines) had a minimal effect on the model. Because data were composed of repeated measures within individuals, we considered its variability by including tag number as a random factor [36]. The GAMM was fit using the gamm4 package [46] in R [47].

## Results

### Snook distribution in Tampa Bay

More net sets captured snook during the spawning season than in the non-spawning season, but snook were sampled in the estuary year-round. A total of 4,920 seine hauls were conducted by the FIM program in Tampa Bay from 1996 to 2012, and 34% of these caught snook ≥350 mm SL. Snook occurred year-round in areas close to shore throughout most of Tampa Bay (Fig. 2) but were concentrated in three areas: on the eastern shore of the main stem of Tampa Bay, on the eastern shore of the mouth of Old Tampa Bay, and on the western shore at the mouth of Tampa Bay (Fig. 2A, 2B). These areas were consequently grouped and defined as a “concentrated” region. In the winter months, fish continued to be present in the concentrated region but in lower numbers and in fewer grids. The number of positive net sets modeled with the binomial CPUE (lowest AIC = 5,884), included the following explanatory variables: year, region (concentrated vs. not concentrated), temperature, and season (spawning vs. non-spawning). However, in those sets that caught snook, the standardized CPUE model with the lowest AIC (5,282) included only year, region, and bottom vegetation. Annual standardized CPUE indicated relatively stable estuarine-wide abundance from 1996–2008, with a decrease in 2009 and the lowest levels in 2010 and 2011 (Fig. 2C, D).

### Inlet spawning site

Spawning activity was confirmed at the inlet spawning site, which fell within the concentrated region identified by the estuarine-wide CPUE analysis (Fig. 2A). Salinities were consistently above 24 ‰ (range: 29.26 to 36.61 ‰) during the spawning season. Based on histological analysis, all tagged females were actively spawning the night of their implantation. These females had either oocytes undergoing late-stage oocyte maturation, indicating spawning was imminent (n = 8), or newly collapsed postovulatory follicles (n = 7), indicating they had just completed spawning prior to capture. In addition, presumed spawning behavior was observed at dusk (1710 to 1915 h) on 31 July 2007, 13 August 2007, and 7 July 2009 in shallow water along the beach on the northern edge of the inlet, corresponding to the northern edge of the core array (Zones 2 and 3). This behavior consisted of “balls” of five to seven fish, typically with one large fish assumed to be female and multiple smaller fish assumed to be males. The fish “rolled” over each other and “flashed” white abdomens as they traveled passively with the current in shallow water (< 2 m) towards the Gulf of Mexico. Observed spawn times ranged from 1700 to 2000 h based on strip-spawning (mean  =  1800 h, n = 4) and ovulating females (n = 7) which were collected over the time period from 1828 to 1942 h.

Some snook moved to the inlet prior to the spawning season. Eleven snook were captured at the inlet in April and May 2007, but no spawning indicators were observed until 22 May when the first running ripe male was sampled. Ovarian biopsies from fish captured in April (n = 4) confirmed that these females were not yet spawning capable. The first spawning female was captured on 4 June.

### Position error and fish survival

Position errors in our array were less than the spatial scale of interest (zone) and detection rates of all control tags were 99.7% or better. Easting position error (mean and SE) of the control tags increased towards the inlet (Zone 6: 10.9 m±0.18 m; Zone 5: 39.2 m±0.75 m; Zone 2: 85.9 m±1.22 m) but positioning errors >200 m were rare in all zones (< 1%). The second spatial metric, mean receiver number per hourly bin, exhibited lower error rates and was used to plot individual movement paths. The pass control tag (placed between receivers 9 and 10) resulted in a position based on the mean receiver number of 9.5 to 10.5±0.05.

All fish survived the implantation process (n = 31), and thirty were relocated after the initial 48 h post-release period (Table 1). On the date of implantation, fish (n = 25 detected) left the spawning site within 4 hours of release. All fish were detected moving towards the estuary and most of them were detected last at receiver 23 (n = 20), just behind the tip of the barrier island (Zone 7).

**Table 1:** Summary of relocations for one-year (n = 20) and multi-year tags (n = 10); a female fish (tag 28) was not relocated after the post-release period and is not included.

Year	Tag	TL (mm)	Sex	DD (A)	First Date (A)	Last Date (A)	TP (A)	RI (A)	DD (SS)	First Date (SS)	Last Date (SS)	TP (SS)	RI (SS)
2007	1	642	M	43	6/4/2007	8/19/2007	77	55.8	29	7/4/2007	8/19/2007	47	61.7
2007	2	640	F	2	6/4/2007	6/8/2007	5	40		.	.	.	.
2007	3	609	M	30	6/5/2007	8/9/2007	66	45.5	10	6/5/2007	8/2/2007	59	16.9
2007	4	570	F	20	6/5/2007	7/21/2007	47	42.6	12	6/21/2007	7/21/2007	31	38.7
2007	5	530	M	28	6/11/2007	8/4/2007	55	50.9	1	6/11/2007	6/11/2007	1	100
2007	6	535	F	18	6/11/2007	8/13/2007	64	28.1	14	6/11/2007	8/13/2007	64	21.9
2007	7	555	M	10	6/14/2007	6/28/2007	15	66.7	3	6/14/2007	6/25/2007	12	25
2007	8	581	M	40	6/14/2007	8/17/2007	65	61.5	23	6/14/2007	8/17/2007	65	35.4
2007	9	547	M	57	6/14/2007	8/15/2007	63	90.5	37	6/14/2007	8/13/2007	61	60.7
2007	10	554	M	81	6/14/2007	9/17/2007	96	84.4	63	6/14/2007	9/16/2007	95	66.3
2007	11	540	M	22	6/14/2007	7/10/2007	27	81.5	12	6/18/2007	7/9/2007	22	54.5
2007	12	546	M	61	6/14/2007	8/20/2007	68	89.7	39	6/14/2007	8/18/2007	66	59.1
2007	13	495	M	72	6/14/2007	9/19/2007	98	73.5	40	6/14/2007	9/18/2007	97	41.2
2007	14	659	F	13	6/30/2007	8/13/2007	45	28.9	2	7/2/2007	7/31/2007	30	6.7
2007	15	792	F	10	6/18/2007	8/30/2007	74	13.5	10	6/18/2007	8/30/2007	74	13.5
2007	16	684	F	7	6/19/2007	8/11/2007	54	13	7	6/19/2007	8/11/2007	54	13
2007	17	618	F	26	6/20/2007	8/22/2007	64	40.6	17	6/29/2007	8/19/2007	52	32.7
2007	18	659	F	17	6/29/2007	7/30/2007	32	53.1	8	6/30/2007	7/30/2007	31	25.8
2007	19	584	F	9	6/19/2007	7/14/2007	26	34.6	3	6/19/2007	7/1/2007	13	23.1
2007	20	575	F	30	6/19/2007	8/12/2007	55	54.5	18	6/19/2007	8/2/2007	45	40
2007	**21**	769	F	14	6/25/2007	8/28/2007	65	21.5	9	7/4/2007	8/28/2007	56	16.1
2007	**22**	660	F	30	6/25/2007	9/15/2007	83	36.1	25	6/25/2007	9/15/2007	83	30.1
2007	**23**	575	F	27	6/4/2007	9/14/2007	103	26.2	6	7/13/2007	9/14/2007	64	9.4
2007	**24**	579	M	79	6/4/2007	9/17/2007	106	74.5	59	6/13/2007	9/12/2007	92	64.1
2007	**25**	689	M	104	6/5/2007	9/20/2007	108	96.3	102	6/5/2007	9/20/2007	108	94.4
2007	**26**	745	F	21	6/5/2007	9/12/2007	100	21	10	7/1/2007	9/12/2007	74	13.5
2007	**27**	759	M	108	6/5/2007	9/20/2007	108	100	54	6/12/2007	9/20/2007	101	53.5
2007	**29**	681	F	101	6/11/2007	9/20/2007	102	99	100	6/11/2007	9/20/2007	102	98
2007	**30**	559	M	29	6/18/2007	7/23/2007	36	80.6	18	6/29/2007	7/22/2007	24	75
2007	**31**	625	M	7	6/25/2007	8/6/2007	43	16.3	4	7/6/2007	8/6/2007	32	12.5
2008	**22**	660	F	23	5/24/2008	9/2/2008	102	22.5	16	5/25/2008	8/29/2008	97	16.5
2008	**23**	575	F	26	5/24/2008	9/2/2008	102	25.5	20	5/24/2008	9/2/2008	102	19.6
2008	**24**	579	M	63	5/11/2008	8/19/2008	101	62.4	53	5/21/2008	8/19/2008	91	58.2
2008	**25**	689	M	178	3/21/2008	9/19/2008	183	97.3	176	3/21/2008	9/19/2008	183	96.2
2008	**26**	745	F	16	3/25/2008	8/1/2008	130	12.3	12	3/25/2008	7/22/2008	120	10
2008	**27**	759	M	176	3/21/2008	9/20/2008	184	95.7	101	3/23/2008	9/19/2008	181	55.8
2008	**29**	681	F	110	5/22/2008	9/19/2008	121	90.9	97	5/22/2008	9/18/2008	120	80.8
2008	**30**	559	M	40	5/19/2008	7/22/2008	65	61.5	36	5/21/2008	7/22/2008	63	57.1
2008	**31**	625	M	1	7/11/2008	7/11/2008	1	.	.	.	.	.	.
2009	**22**	660	F	30	5/18/2009	9/6/2009	112	26.8	28	5/18/2009	8/25/2009	100	28
2009	**23**	575	F	72	4/26/2009	9/17/2009	145	49.7	27	6/6/2009	9/5/2009	92	29.3
2009	**24**	579	M	76	5/17/2009	9/7/2009	114	66.7	65	5/17/2009	9/7/2009	114	57
2009	**25**	689	M	180	3/21/2009	9/20/2009	184	97.8	180	3/21/2009	9/20/2009	184	97.8
2009	**26**	745	F	*	4/1/2009	*	*	*	*	6/8/2009	*	*	*
2009	**27**	759	M	178	3/21/2009	9/20/2009	184	96.7	145	3/23/2009	9/18/2009	180	80.6
2009	**30**	559	M	*	5/8/2009	*	*	*	*	5/8/2009	*	*	*

TL = total length; DD = number of dates detected; TP = total period of detection; RI = Residence index; A = array; SS = spawning site (Zones 2 and 3). First date refers to the date of implantation in 2007 and first relocation date in subsequent years. Asterisks denote fish that died in 2009.

### Site fidelity and residence times

Fish exhibited both intra-seasonal and inter-annual site fidelity, repeatedly moving to and from the inlet spawning site within the spawning season. Most fish (n = 28) detected in 2007 were relocated on multiple dates in the spawning site (Table 1) and all but one were detected for less than 24 h on any given date (mean number of hours detected per date: 5.6±0.2 SE). Mean DD_SS_ of these fish was 39 d and ranged from 1 to 102 d. Ten fish with multiple-year tags were detected in 2007. In 2008, nine of these were detected and eight fish returned to the spawning site. In 2009, seven of these fish again returned and all seven fish were relocated in the spawning site.

**Figure 3:**
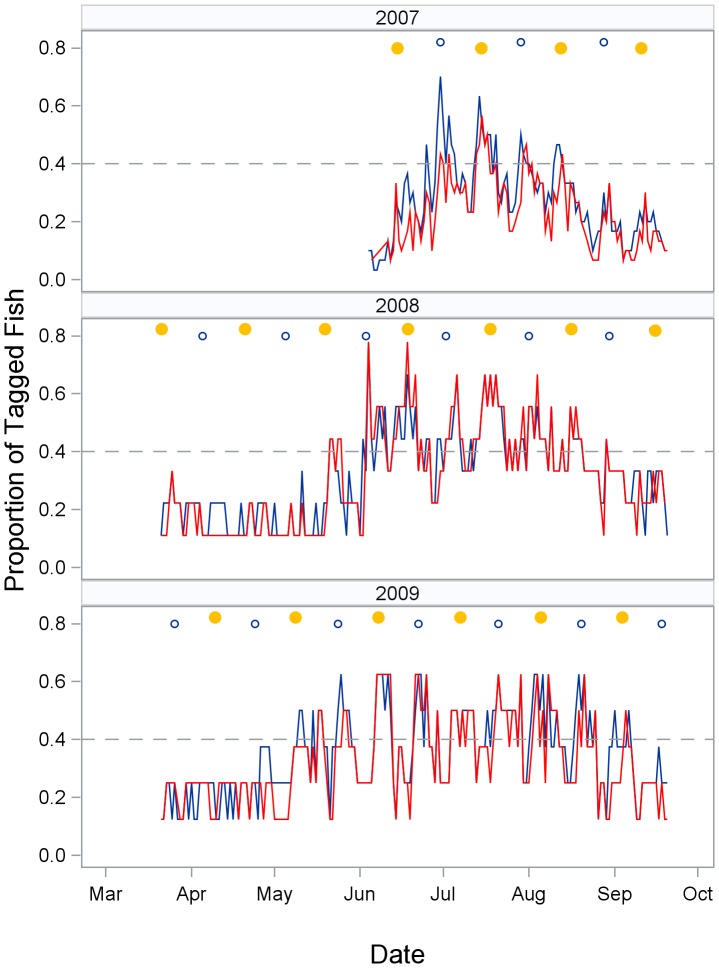
Individual relocation periodicities. First and last date, inter-quartile range (25–75%), mean (circle) and median (line) of relocation dates. Tag numbers 1–20 had one year batteries and could only be relocated in 2007. The range of relocation dates are presented by year and by relocation in non-spawning site zones (Zones 4–7) or in the spawning site, Zones 2 and 3. Males are represented by blue and females by red. Reference lines indicate 22 May, the first date active spawners were observed and 15 September, the presumed end of the spawning season. Multi-year tags were active from 22 March to 20 September. Asterisks indicate fish which died or lost their tags and thus do not represent the full period they might have been present.

Most fish were detected in the array over a range of dates more restricted than the period the tags were active (22 March through 20 September), but a few fish were detected over longer time periods (Fig. 3, Table 1). In 2008, the majority of fish were first detected both in the array and in the spawning site in mid-May. However, three fish were detected in March: two males (tags 25 and 27) were consistently relocated throughout the period that the tags were active and a female (tag 26) was detected one day in March and then not again until May. These patterns remained consistent in 2009. Even though V9 tags were active through October 2007, the mean departure date for all three years was in mid-August (2007: 16 August; 2008: 23 August; 2009: 19 August). Because fish were implanted in June 2007, mean residence time (TP) in that year was 65 d (n = 30), compared to approximately four months in subsequent years (2008: 122 d, n = 9; 2009: 110 d, n = 7), considerably less than the 184 d that tags were actively transmitting. As mentioned above, two males exhibited a different temporal pattern with TPs >180 d. Two recaptures of acoustically tagged fish by anglers in late fall and winter also indicated some fish remain in the area over longer time periods. Tag 18 (F) was captured behind the barrier island near receiver 23 on 13 October 2009 and tag 31 (M) was captured at Sawyer Key on 12 January 2008 (Fig. 1).

**Figure 4:**
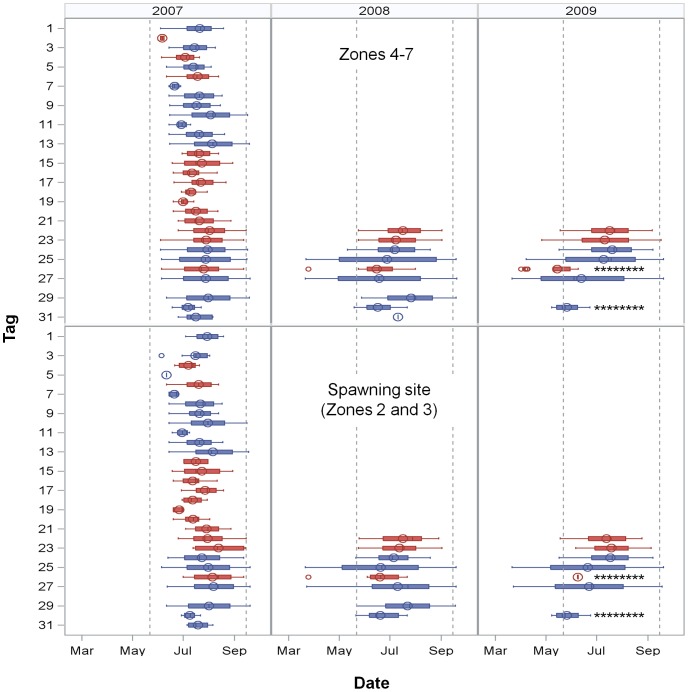
Lunar and seasonal pattern of fish relocations. Proportion of tagged fish detected in spawning site zones (red) and non-spawning site zones (blue) by date and year. The full moon phase is indicated by yellow circles and the new moon by open circles. A reference line is marked at the 0.4 relocation rate to help assess seasonal trends in relocations. The similarity in temporal patterns for spawning site and non-spawning site zones indicates many snook leave the array area between spawning events.

Snook exhibited intra-seasonal fidelity to the inlet spawning site but had varying relocation rates. RI_SS_ exhibited a strong correlation with RI_A_ (Pearson correlation coefficient = 0.90, n = 38, P<0.0001). The proportion of fish detected repeatedly spiked on dates near the new and full moons and then fell below 0.4 in between (Fig. 4). This temporal pattern in relocation rates was similar for both spawning site zones and non-spawning site zones, indicating many fish moved to and from the spawning site over a larger spatial scale than monitored by the array. In 2007, the proportion of fish relocated within the array on any given date ranged from 0.03–0.70 (n = 1 to 21) with the most fish detected on 30 June. In 2008 and 2009, the relocation patterns were similar, with the greatest number of fish detected in June, July, and August and on dates near the new and full moons (Fig. 4). However, seasonal patterns differed somewhat in their duration and peak spawning period. In both 2007 and 2008, strong peaks occurred early in the spawning season, with relocation rates decreasing as the season progressed. In contrast, in 2009, peak relocation rates remained relatively constant over a more extended spawning season. Although both sexes had relatively high relocation rates, male RI_A_ values were significantly greater than female values (Mann-Whitney Test U = 454, *DF* = 1, *P*<0.001), as were male RI_SS_ values (Mann-Whitney Test U = 398; *P*<0.001, DF = 1; Table 1).

### Movement patterns

**Figure 5:**
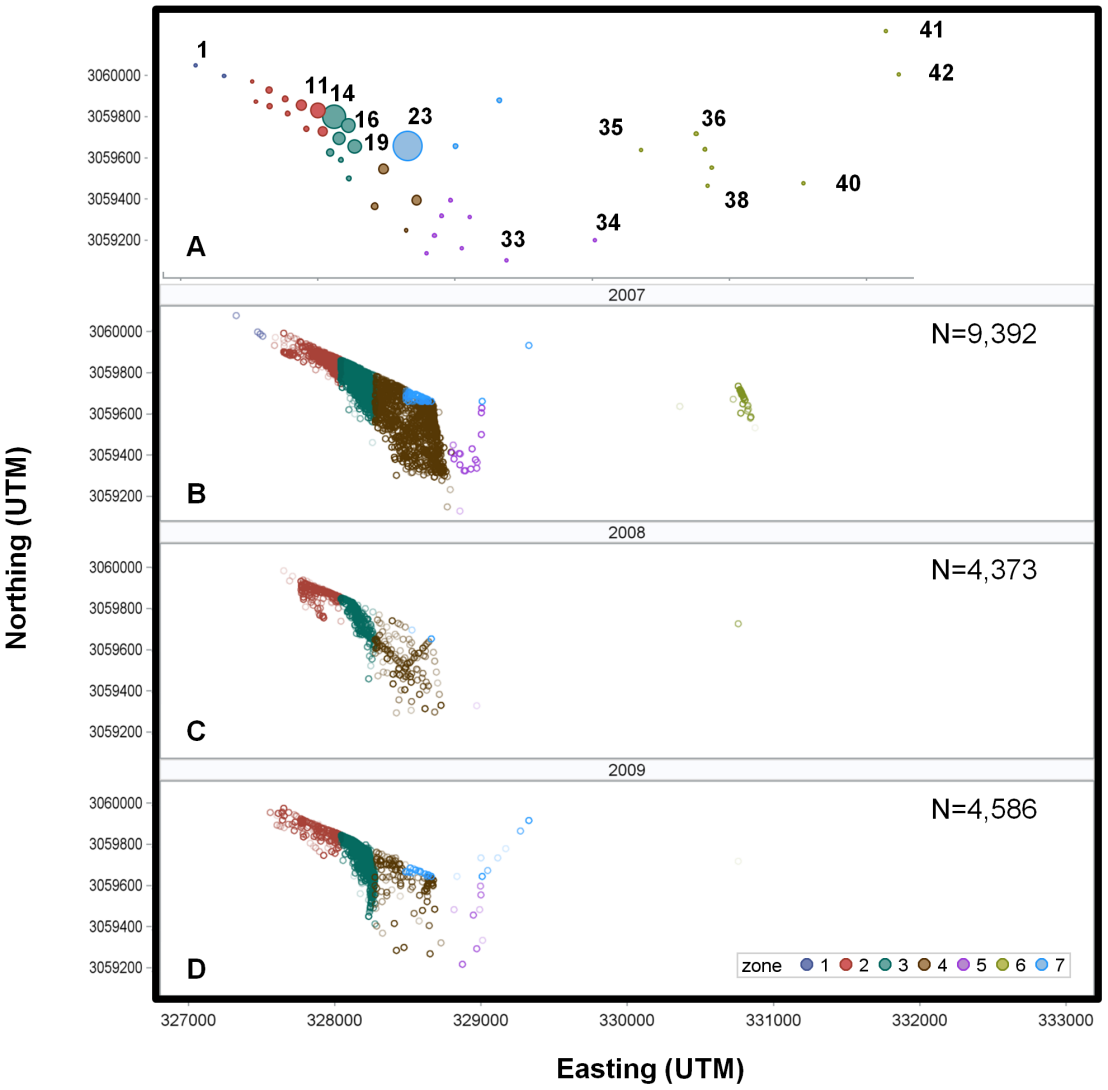
Spatial pattern of detections and fish positions. The top panel indicated receiver locations and zones. (A) Proportion of relocations by receiver (size corresponds to frequency of detections); and positions plotted by zone in: (B) 2007 (n = 31 fish); (C) 2008 (n = 9 fish); and (D) 2009 (n = 7 fish). Positions are color-coded to represent zone.

**Figure 6:**
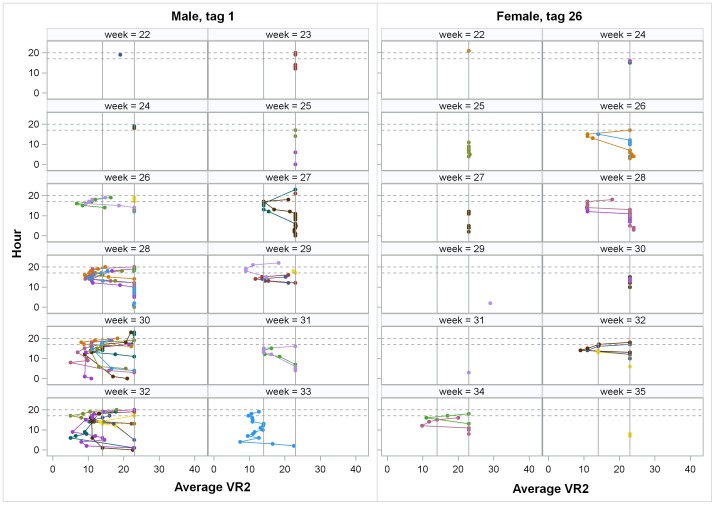
Individual movements. Relocation paths by week for one male and one female in 2007. Colors represent the movement path for a given date. Position is based on the average receiver number within an hourly time bin. Horizontal reference lines reflect hours associated with spawning activity (17:00–20:00 h) and the vertical reference line indicates receiver 23 corresponding to a refuge area.

The tagged fish exhibited similar spatial patterns over all three years, with snook detected most frequently on receivers near the barrier island and in Zones 2, 3, 4, and 7 (Fig. 5). Receiver 23, behind the barrier island in Zone 7, had the highest proportion of detections (0.32; Fig. 5A) with receiver 14 (in the spawning site, Zone 3) having the second highest (0.20). Additional receivers accounting for more than 2% of total detections were: receivers 21 and 24 in Zone 4 (cumulative 0.07), detecting fish as they moved between the inlet spawning site and the area behind the barrier island; receivers 19 (0.06) and 17 (0.07) in Zone 3, along the eastern edge of the core array; and receivers 11 (0.08) and 9 (0.03) in Zone 2 on the northern edge of the spawning site (Fig. 5A). For zones with the greatest relocations (Zones 2, 3, 4, and 7), detections were not equally distributed nor consistently affiliated with zones with the greatest number of receivers (*Χ* ^2^  = 3186.8, *P*<0.0001, *DF* = 3; Table 2). Individual daily movement paths helped explain these patterns, as most individuals (n = 28) commonly moved from the area behind the barrier island (detected at receiver 23) to the inlet spawning site and back (Fig. 6). The area monitored by receiver 23 appeared to act as a refuge, as fish were commonly detected at this receiver for several weeks after implantation and during hours not associated with spawning. On some dates, movement to the inlet was associated with spawning times, but on other dates fish moved to the inlet in the early morning and did not leave until 2000 h or later.

**Table 2:** Table of relocations (number and proportion) by year and zone.

Year	Zone							
	1	2	3	4	5	6	7	Total
2007	11	505	622	662	15	28	883	2726
	0.004	0.185	0.228	0.243	0.006	0.010	0.324	
2008	0	304	448	321	1	4	289	1367
	0.000	0.222	0.328	0.235	0.001	0.003	0.211	
2009	0	267	432	123	8	1	419	1250
	0.000	0.214	0.346	0.098	0.006	0.001	0.335	
Total	11	1076	1502	1106	24	33	1591	5343
	0.002	0.201	0.281	0.207	0.005	0.006	0.298	

Relocations are based on one hour time bins. Zones 2 and 3 are associated with the spawning site.

**Figure 7:**
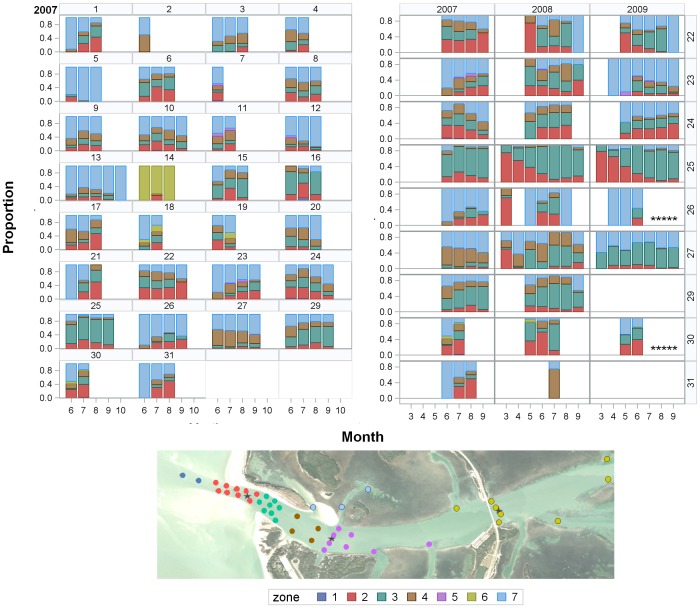
Spatio-temporal patterns of individual fish. Proportion of detections by zone and month for individual fish in 2007 (on the left), tag number is labeled above each panel; and fish with multi-year tags (on the right) over the months tags were active, tag number is labeled to the right of the three-year panels. Asterisks indicate fish which died or lost their tags and, thus, do not have data for the remaining months.

Individual spatio-temporal patterns indicated several contingents. Although most fish were detected in zones associated with the barrier island, one fish (tag 14) exhibited a spatial pattern differing from the others, being predominantly relocated in Zone 6 (Fig. 7). The months of detection also differed by individual, ranging from one to five months in 2007 and one to seven months in subsequent years. As previously mentioned, two fish (tags 25 and 27) exhibited patterns indicating they were resident in the area over a greater time period than the spawning season while the remaining fish appeared to move to the area during months associated with the spawning season. The relative proportion of detections in the spawning site (Zones 2 and 3) versus the refuge area (Zone 7) differed over time and between individuals.

### Factors affecting presence at the spawning site

The temporal pattern of detections at the spawning site suggested abundance at this location is driven by reproductive behavior, and the number of fish spawning at this site varies within the spawning season. In the GAMM model for all fish in 2007, significant predictor variables were sex (*p* = 0.000153), lunar phase (*p*<0.0001), and day of the year (*p*<0.0001). The probability of occurring at the spawning site increased in peak spawning months (June, July), on dates near the new and full lunar phases, and was greater for males than females. Sex-specific size, however, was not significant. The GAMM model for fish (n = 7) detected over multiple years had the same significant variables: sex (*p* = 0.00489), lunar phase (*p*<0.0001), and day of the year (*p*<0.0001).

## Discussion

There is increasing awareness that population structure is often complex in marine and diadromous species, and that this structure has important implications for management [48]–[50] For most species, little is known about the process of spawning site selection, the temporal scale of site fidelity, or how fine scale spatio-temporal behavior may impact reproductive success [33], [51]. However, failing to account for this complexity can lead to the extirpation of stock components [52], [53] that in turn affect productivity [54], [55], stability and resilience [49]. Much of the seminal work on population complexity and contingents has been conducted on diadromous fishes, as they exhibit predictable migrations between spawning and foraging habitat that need to be understood for effective management [56], [57].

### Facultative catadromy

Catadromous species are defined as spending much of their lives in freshwater but migrating to marine habitats to spawn with little overlap between their freshwater foraging habitat and their marine reproductive habitat [58]. Common Snook are defined as catadromous, obligate marine spawners [19], [59] with the following spatial components to their life cycle: spawning in high salinity areas near passes and beaches, with variable rates of site fidelity, estuarine larval retention and oligo- and mesohaline wetland nursery habitat, with adults foraging and overwintering in freshwater [15], [60]. However, the spatio-temporal pattern of Common Snook is more complex than this paradigm [22], [29]. Similar to others, our study demonstrated some snook remain in the estuary year-round [22]. In addition, we showed that although there were fewer snook in the estuary in the winter, they remained concentrated in the same areas as they were during the spawning season.

Snook exhibit behavioral contingents, as seen in other diadromous fishes [11], [49], with variable habitat use (freshwater versus estuarine) and temporal patterns associated with that use. Common Snook showed seasonal shifts in abundance between estuarine and freshwater habitat in Charlotte Harbor (approximately 130 km south of Tampa Bay), with abundance greatest in the estuary in the summer and in the rivers in the fall [22]. This, in conjunction with our results, suggests there is a contingent that demonstrates more typical catadromous behavior while another remains in the estuary throughout the year [22], [29]. In addition, in a recent telemetry study tracking Common Snook in the Caloosahatchee River, which flows into Charlotte Harbor, only 40% of the fish left the river during the spawning season [23]. Thus, there may be another contingent that remains in the rivers over extended periods, opting out of one or more reproductive cycles (i.e., skip spawning).

These results are similar to other species previously believed to be obligate catadromous, such as American eels, *Anguilla rostrata* [61], the European eel, *Anguilla anguilla,* the Japanese eel, *Anguilla japonica,* [62] and the European flounder, *Platichthys flesus* [63]. Each of these species exhibits plasticity in their spatial life histories similar to that of Common Snook. Common Snook is a subtropical species and Florida is at the northern extent of its range [64]. Catadromy is more common in the tropics [58], but has been shown in eels to shift from obligate to facultative with increased latitude. This is hypothesized to be due to the tendency in the tropics for freshwater habitats to be more productive than oceans [65], with the opposite trend as latitudes increase [61]. Thus, we hypothesize that Common Snook may have adapted to use a wider range of habitats at this northern range, given high estuarine productivity, resulting in facultative catadromy rather than obligate catadromy.

### Spawning site selection and fidelity

Common Snook are reported to require salinities >24 ‰ to activate sperm cells, maintain buoyancy of fertilized eggs [19], [59], and spawn at passes and the beaches. In Tampa Bay, Common Snook concentrated during the spawning season in areas primarily along the eastern shore. Salinities in these areas often exceeded 24 ‰. In contrast, salinities at the inlet spawning site were consistently above 24 ‰. The only confirmed spawning sites within the study area are the inlet spawning site and the lower eastern shore of Tampa Bay [66]. Given the close correlation between Common Snook concentration areas and spawning sites on the east coast of Florida [25], we hypothesize that these areas of concentration correlate with spawning activity. All of the high-concentration areas had relatively pristine shores with seagrass and mangrove habitat, both important to Common Snook [29], [67]. In addition, the area along the eastern shore is in close proximity to two rivers, the Little Manatee River and the Manatee River. Common Snook in these areas may spawn over a more restricted period than at the inlet site (i.e., only when salinities are high enough) or they may be able to spawn in a wider range of salinities than previously believed. Maternal environment has been shown to affect salinity thresholds needed for egg buoyancy in other species [68] and similar adaptability to variable salinity habitats has been exhibited by the European Flounder [63]. If these areas are spawning habitat, then spawning site selection by Common Snook may involve trade-offs between habitat with consistently high salinity, allowing for more reproductive events within a year, and habitat that is less predictable but provides greater refuge and/or feeding opportunities.

However, without sampling fish in these areas for reproductive state, we cannot rule out the possibility that snook migrate from these areas to passes and beaches to spawn. We demonstrated that pulses in numbers of fish at the spawning site occurred within the spawning season. The similarity in these pulses for both spawning site and non-spawning site zones (Fig. 4) indicated many snook leave the area monitored by our array between spawning events. Individual snook can undertake long within-spawning season migrations, as an individual fish from the east coast of Florida was shown to leave a pass spawning site, move to a low-salinity area (14.6 km away), and later return to the spawning site [24]. From an energetics viewpoint, the concentration area at the head of Tampa Bay seems the least likely to support this type of behavior as fish would need to migrate almost 30 km to a pass spawning site. However, further research is needed to determine the range of spawning habitat and large scale movements.

At the inlet spawning site, detection patterns suggested that most fish move to this area during or just prior to the spawning season, similar to what has been reported for the east coast of Florida [25]. Most fish were first located in the spawning site in mid-May, with peak numbers occurring in June and July, and a mean date of departure in mid-August. These temporal patterns dovetail well with migration patterns seen in fish migrating from rivers during the spawning season with a mean departure date of 1 June, mean return date of 18 August, and a total mean migration period of 78 d [23]. We found a longer mean residence time at the spawning site of 112 d. However, if the two fish with residence times >180 d are removed, mean residence time at the inlet spawning site is comparable (86 d) and indicates individual spawning periods are shorter than the reported population spawning season (mid-April through mid-September, approximately 150 d).

Common Snook exhibited spawning site fidelity to the inlet spawning site. Spawning site fidelity simply reflects consistent spawning site selection over time and is often assumed to occur at the lifetime scale. However, for iteroparous, multiple-batch spawners, spawning site selection and fidelity can vary over several temporal scales: lifetime, annual, and inter-annual (i.e. within the spawning season). For all species, there can also be variability in this behavior at the population, contingent, or individual scales. Understanding these processes is considered a major challenge in marine ecology today [6]. Several hypotheses have been suggested to explain spawning site fidelity, including natal homing and learned behavior through “spawning groups” formed at first maturity or by following the behavior of older fish [26], [69]. However, given that snook were not common at this site until 2006 (Lowerre-Barbieri et al. unpubl. data) and the relatively large size of Common Snook at this site, these hypotheses do not explain the behavior observed in this study.

The inlet spawning site described in this study also supports a Spotted Seatrout, *Cynoscion nebulosus*, spawning aggregation [38] that has been studied since 2001. Common Snook were observed at this inlet site in 2006, after Spotted Seatrout were negatively impacted by an unusually persistent red tide (*Karenia brevis*) event in 2005 [30], [33]. Because red tide typically cannot survive in salinities less than ∼24 ‰, not all areas in Tampa Bay were equally affected [70]. Spotted Seatrout juvenile abundance in Tampa Bay was significantly lower in 2005 and 2006 than in previous years. In contrast, Common Snook juveniles remained stable in 2005, with a peak in abundance in 2006 [30]. This has important implications, suggesting that Common Snook are more capable of moving to freshwater refuge areas than Spotted Seatrout and that Common Snook continued to spawn successfully in 2005, while Spotted Seatrout did not [33].

The dynamics between these two species may have played a role in Common Snook selecting this spawning site. The high spawning site fidelity at small spatial scales previously reported suggested that Common Snook exhibited a rigid spatial structure and might be less resilient to disturbance events and fishing pressure because of this rigidity [26], [27]. However in this study, we observed adult Common Snook colonize a new spawning site and exhibit subsequent inter-annual and intra-annual fidelity to it. At larger scales, increased spatial distributions are typically assumed to be correlated with times of greater abundance [4], but adult Common Snook abundance in Tampa Bay was stable from 2004–2008. Thus, it appears that more Common Snook moved to this site to spawn when the site supported fewer Spotted Seatrout. The FIM data indicated Common Snook were concentrated in the areas surrounding the spawning site, and thus had proximity to this site. However, the mechanism by which Common Snook would be attracted to this location in high numbers, over such a relatively short time remains unknown.

Once established at this site, Common Snook exhibited spawning site fidelity within the spawning season and over the three-year period. The environmental or social cues resulting in the initial selection of this spawning site presumably also cued annual site fidelity. However, little is known about within season site fidelity or what might drive this behavior, as environmental cues will change as the season progresses [33], [71]. However, this behavior affects the efficacy of traditional methods used to estimate annual fecundity in indeterminate species and has important implications for our understanding of population structure. Individual movement paths showed fish were not resident in the spawning site, but rather moved to and from the site with greater movement to the spawning site in some weeks than others (Fig. 6). This behavior violates the assumption of no immigration or emigration needed for traditional methods to assess spawning frequency [72]. There is also growing awareness that, similar to our results, many individuals do not spawn throughout the duration of the observed population spawning season [71], [73] affecting our ability to estimate annual fecundity. Individual variability in spawning periods also has important implications for population structure. At the population scale, Common Snook exhibit an extended spawning season from mid-April through mid-September, resulting in a wide range of birthdates. Birthdate, in turn, will affect life history traits and can be the source of contingent behavior, as the growth environments associated with early born versus late-born individuals will differ [74], [75].

### Fine scale movements at the spawning site

The ability to couple physical with biological understanding in model simulations provides insight into factors affecting connectivity and recruitment success and these bio-physical models are evolving to address ecological variability including small scale oceanographic features and larval behaviors [76]. However, fine scale spatio-temporal reproductive data are often not available. Reproductive timing and spawning site selection determine the environment a fertilized egg first encounters and thus affects the hydrologic and predator environments and its probability of survival [73]. Spawning activity of Common Snook varied over diel, weekly, and seasonal temporal scales, with spawning observed from 1700 h to 2000 h, increasing with the new and full lunar phases and in June and July. There is a need for future research on Common Snook juveniles to integrate these patterns in spawning activity with juvenile survivorship. Spawning activity also varied with sex, with males returning to the spawning site more frequently than females. Similar gender differences in reproductive behavior are increasingly reported [33], [77] and although not typically presented as contingents, seem to fall within this definition.

## Conclusions

Although our understanding of population structure has greatly improved in the past decade, there continues to be a need to conduct directed research on spatio-temporal reproductive behavior and to integrate these results into larger conceptual models. Snook in southwest Florida exhibit complex spatio-temporal behavior, utilizing a range of habitats and exhibiting multiple behavioral contingents. Their spatial distribution during the spawning season and ability to move long distances to freshwater habitat confers greater resilience to disturbance events occurring in high-salinity habitats, such as red tide events. However, the long-held belief that river systems provide thermal refuge is not supported, given the observed decreases in adult abundance after an extreme cold event in January 2010 in Tampa Bay and Charlotte Harbor [64].

Spawning site selection and fidelity underlie our ability to manage marine and diadromous fish sustainably. Spawning behavior is the starting point for population structure and will determine whether eradicated spawning components can recover. Much of our understanding of these processes is based on early life history research, but with acoustic telemetry we have the additional ability to observe movements associated with spawning *in situ*. Thus to understand the complexities of a species' life cycle over space and time, there needs to be greater integration of reproductive spatio-temporal behavior into the models of marine and diadromous population structure and dispersal.
