# Correction: Spawning Site Selection and Contingent Behavior in Common Snook, *Centropomus undecimalis*

**DOI:** 10.1371/journal.pone.0113544

**Published:** 2014-11-10

**Authors:** The PLOS ONE Staff

The images for Figures 3 and 4 are incorrectly switched. The image that appears as Figure 3 should be Figure 4, and the image that appears as Figure 4 should be Figure 3. The figure legends appear in the correct order.

**Figure 3:**
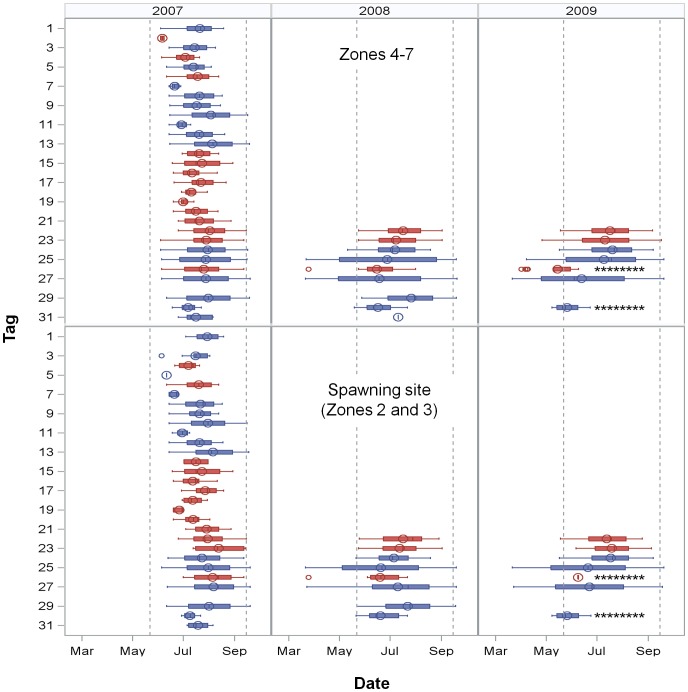
Individual relocation periodicities. First and last date, inter-quartile range (25–75%), mean (circle) and median (line) of relocation dates. Tag numbers 1–20 had one year batteries and could only be relocated in 2007. The range of relocation dates are presented by year and by relocation in non-spawning site zones (Zones 4–7) or in the spawning site, Zones 2 and 3. Males are represented by blue and females by red. Reference lines indicate 22 May, the first date active spawners were observed and 15 September, the presumed end of the spawning season. Multi-year tags were active from 22 March to 20 September. Asterisks indicate fish which died or lost their tags and thus do not represent the full period they might have been present.

**Figure 4:**
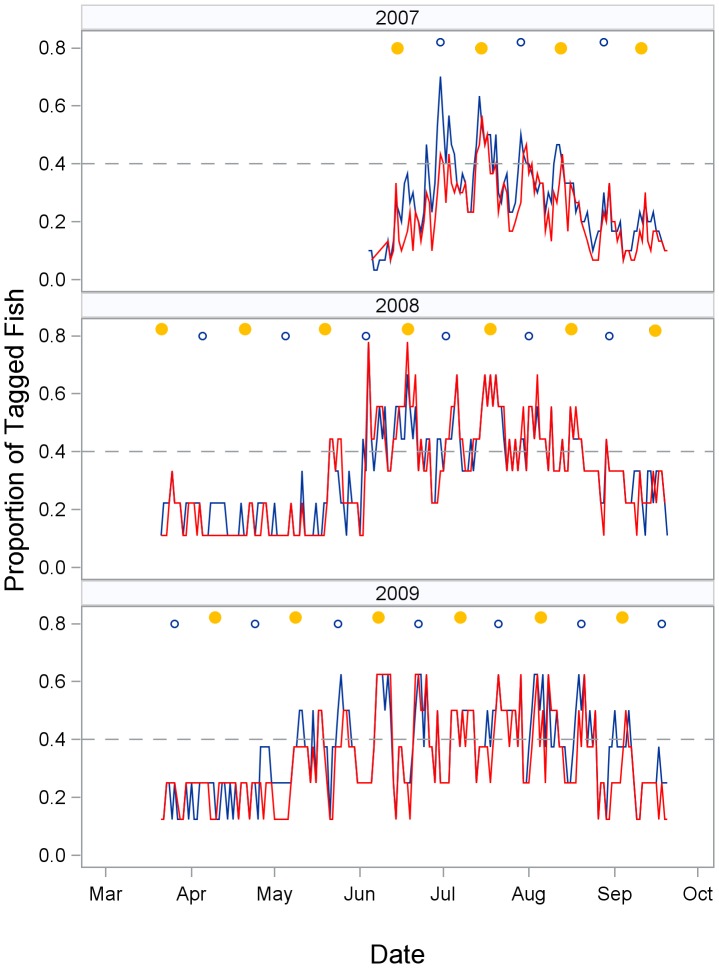
Lunar and seasonal pattern of fish relocations. Proportion of tagged fish detected in spawning site zones (red) and non-spawning site zones (blue) by date and year. The full moon phase is indicated by yellow circles and the new moon by open circles. A reference line is marked at the 0.4 relocation rate to help assess seasonal trends in relocations. The similarity in temporal patterns for spawning site and non-spawning site zones indicates many snook leave the array area between spawning events.
